# Pelizaeus-Merzbacher disease in siblings

**DOI:** 10.4103/1817-1745.76122

**Published:** 2010

**Authors:** Amit Mittal, Baljeet Maini, P. D. Sharma, Amit Aggarwal

**Affiliations:** Department of Radiodiagnosis, MM Institute of Medical Sciences and Research, Mullana, Ambala, Haryana, India; 1 Department of Pediatrics, MM Institute of Medical Sciences and Research, Mullana, Ambala, Haryana, India

Dear sir,

Pelizaeus-Merzbacher’s disease (PMD) is a rare X-linked inherited disorder affecting myelination of the central nervous system. Pathologically, PMD, in contrast to other leukodystrophies like metachromatic leukodystrophy, adrenoleukodystrophy and multiple sclerosis, is a dysmyelinating rather than a demyelinating disorder. In demyelinating disorders, myelin is formed, deposited around the axons and then destroyed later. In dysmyelinating disorders such as PMD, normal myelination does not occur.[1]

Two brothers (11-years-old and 10-years-old) presented to the pediatrics department with complaints of delayed development, ataxia, mental retardation, language impairment and difficulty in walking, which was progressive. They were born at full-term gestation without any adverse antenatal or perinatal course. Both patients attained head holding at 3 years. There was also history of nystagmus since they were 1 year old. There was no significant family history.

On detailed physical and neurological examination, there was mental retardation, vertical peduncular nystagmus, hypereflexia and spasticity in both upper and lower limbs (more in the lower limbs) in both siblings, with a positive Babinski sign. They could walk with support and there was severe ataxia. There also was language impairment with dysmetria in the sibling. The upper limbs were normal. Their auditory and visual somatosensory-evoked potentials were normal.

**Figure 1:**
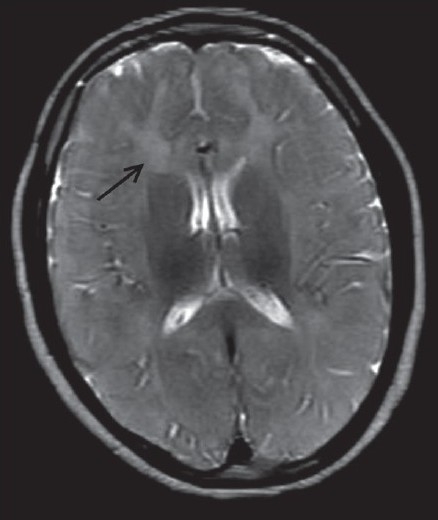
Magnetic resonance imaging T2W sequence in a 11-year-old child showing diffuse white matter hyperintensity (arrow) in the supratentorial white matter

**Figure 2:**
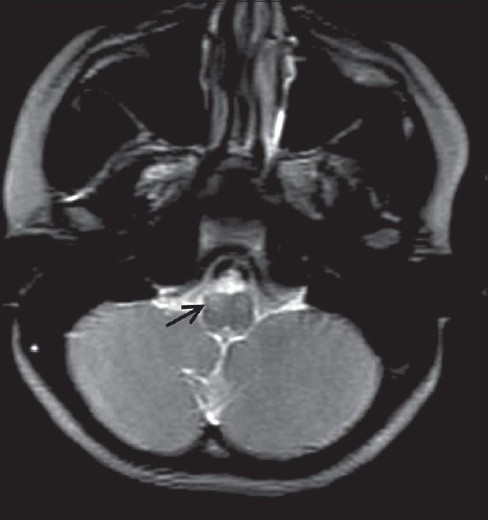
Magnetic resonance imaging T2W axial sequence in a 11-year-old child showing white matter hyperintensity (arrow) in the medulla

**Figure 3:**
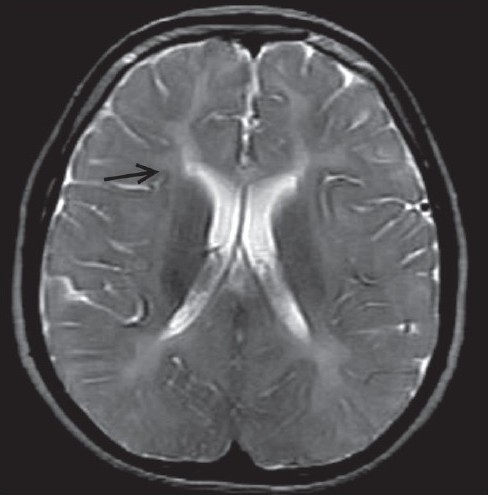
T2W axial magnetic resonance image in a 10-year-old child showing bilateral symmetrical subtle hyperintense lesions (arrow) in the white matter

**Figure 4:**
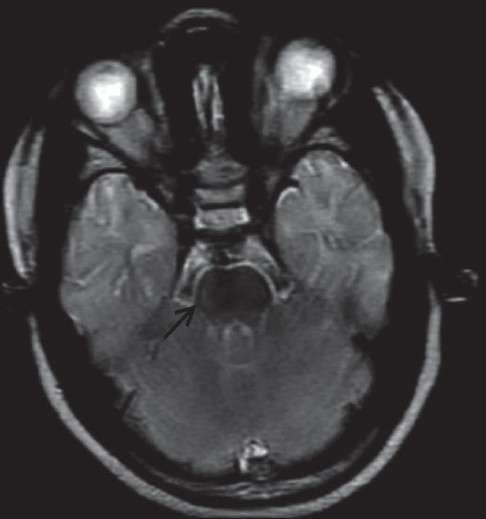
T2W axial magnetic resonance image in a 10-year-old child showing subtle hyperintense lesions in the pontine white matter (arrow)

Chest radiograph and all routine hematological investigations were normal. The patients were sent for a magnetic resonance imaging (MRI) of brain. MRI was carried out on 0.2 tesla Signa (GE systems, Waukesha, Wisconsin, USA) MRI with T2W, T1W and FLAIR sequences in all three planes. On MRI, there was diffuse and symmetrical subtle high signal intensity on the T2W sequence in the bilateral supratentorial white matter, brainstem and cerebellum in both siblings [Figure 1–4]. Thalami, basal ganglia and corpus callosum were normal.

Thus, all these clinical and radiological findings were diagnostic of PMD.

PMD and X-linked spastic paraplegia type 2 (SPG2)are two sides of the same coin. Both arise from mutations in the gene encoding myelin proteolipid protein 1 (PLP1). The disease spectrum for PMD and spastic paraplegia type 2 is extraordinarily broad, ranging from a spastic gait in the pure form of spastic paraplegia type 2 to a severely disabling form of PMD featuring hypotonia, respiratory distress, stridor, nystagmus and profound myelin loss. The frequency is estimated to be 1 case per 100,000–1,000,000 population.[2]

PMD typically affects males, but female heterozygotes can be clinically affected, especially those who carry alleles that are relatively mild in males. Based on the time of onset and the clinical severity, PMD has traditionally been divided into four categories: classic, connatal, transitional and adult forms. The classic and connatal forms are the most common. Classic PMD has its onset during late infancy. Early symptoms include nystagmoid, dancing or trembling eye movements and delayed motor development followed by involuntary movements and spasticity. The course is usually protracted and it is often misdiagnosed as cerebral palsy. Connatal PMD is a rarer and more severe variant that begins at birth or in early infancy and has a more severe clinical course. Abnormal nystagmoid eye movements, extrapyramidal hyperkinesia, spasticity, optic atrophy and seizures also occur during the early stage.[3,4] Both our patients presented with the classic form of PMD as both had developed head holding and could walk, although with ataxia and spastic gait. There was no history of seizure or signs of optic atrophy.

Severe clinical syndromes (the connatal form) are typically caused by missense and other small mutations that affect critical positions in PLP1, whereas the milder spastic paraplegia syndrome is caused by mutations that presumably affect less-critical regions of the protein. The most common mutations that cause PMD are duplications of a region of the X chromosome that includes the entire PLP1 gene.[1,5]

MRI is a useful method for assessing the dysmyelination of the cerebral white matter in PMD. MRI can show a hypomyelination pattern, *i.e*., reversal of the white matter signal intensity on T1- and T2-weighted images. In PMD, MR images generally show either diffuse or patchy (tigroid) T2 hyperintensity in the cerebellar, brain stem and supratentorial white matter. This appearance is believed to be the result of the lack of formation of myelin (hypomyelination or dysmyelination). Diffuse, confluent involvement is usually seen in the severe connatal form, whereas the tigroid pattern is more common in the patients with SPG2. Atrophy and decreased white matter volume may also occur.[6] In our both cases, there was evidence of diffuse white matter T2W hyperintensity in the supratentorial white matter and brainstem white matter. There was mild cerebral atrophy.

Recently, few reports have described proton MRI spectroscopy findings in this disease, with diffuse or focal reductions in *N*-acetylaspartate in the affected white matter. These reductions seemed to be consistent with axonal damage. In addition, mild increases in choline and creatine levels were observed, which may have been due to astrocytic changes.[7] We cannot perform spectroscopy because of equipment limitation.

In conclusion, the diagnosis of PMD should be considered in neonates or children with such clinical features and MRI findings.
